# Dr. McLaughlin’s Paper

**Published:** 1888-01

**Authors:** 


DR. McLAUGHLIN'S PAPER, in the present issue, embraces his views on contagion and septic poisoning, as expressed in the discussion of Dr. Hill's paper, (herewith published) which was read at the last meeting of the *Austin District Medical Society.* We had intended to publish it as part of the discussion, but in view of its length and value, and the importance of the subject Dr. McLaughlin was prevailed upon to prepare it in the shape in which it appears herewith. It will be read with interest, as it contains views altogether new and original, and they are presented in a manner both forcible and concise.

